# Addressing common inferential mistakes when failing to reject the null-hypothesis

**DOI:** 10.12688/f1000research.158434.2

**Published:** 2025-02-25

**Authors:** Amand Schmidt

**Affiliations:** 1 Department of Cardiology, University of Amsterdam, Amsterdam Zuidoost, 22660, Netherlands Antilles; 2 University College London Faculty of Population Health Sciences, London, England, UK; 3 Department of Cardiology, Utrecht University, Heidelberglaan, 3584 CX, Netherlands Antilles; 4 UCL British Heart Foundation Research Accelerator, London, Chenies Mews, WC1E6HX, UK

**Keywords:** Statistical inference; null-hypothesis; equivalence testing, statistical power; accuracy.

## Abstract

Failure to reject a null-hypothesis may lead to erroneous conclusions regarding the absence of an association or inadequate statistical power. Because an estimate (and its variance) can never be exactly zero, traditional statistical tests cannot conclusively demonstrate the absence of an association. Instead, estimates of accuracy should be used to identify settings in which an association and its variability are sufficiently small to be clinically acceptable, directly providing information on safety and efficacy. Post-hoc power calculations should be avoided, as they offer no additional information beyond statistical tests and p-values. Furthermore, post-hoc power calculations can be misleading because of an inability to distinguish between results based on insufficient sample size and results that reflect clinically irrelevant differences. Most multiple testing procedures unrealistically assume that all positive results are false positives. However, in applied settings, results typically represent a mix of true and false positives. This implies that multiplicity corrections do not effectively differentiate between true and false positives. Instead, considering the distributions of p-values and the proportion of significant results can help to identify bodies of evidence unlikely to be driven by false-positive results. In conclusion, rather than attempting to categorize results as true or false, medical research should embrace established statistical methods that focus on estimation accuracy, replication, and consistency.

## Background

Statistical tests and p-values are used to estimate the compatibility of the available data with a specific null-hypothesis. While statistical tests and p-values are strongly embraced by applied researchers, statistical science has raised important concerns about their interpretability, which may lead to incorrect statements about the presence, absence, or importance of an association. ^1^ This has led some researchers to suggest the replacement of p-values with alternative metrics, such as the S-value (i.e., S for surprise). ^2, 3^ The current manuscript attempts to provide an accessible overview of these voiced concerns, particularly focusing on the appropriate interpretation of “non-significant” results when a p-value or statistical test does not provide sufficient reason to reject the posed null-hypothesis. In line with previous guidance in this area, this manuscript suggests moving away from categorizing results as *True* or *False*, instead suggesting research focusses on obtaining sufficiently accurate results on potential benefits and harms.

As an illustrative example, we will consider results from the VOYAGER PAD ^4^ trial, which randomized patients with peripheral artery disease (PAD) to twice-daily rivaroxaban (2.5 mg) or a placebo, evaluating differences in the incidence of ischemic cardiovascular disease. The reported hazard ratio (HR) for rivaroxaban was 0.85 (95%CI 0.76; 0.96), tested against a null-hypothesis HR of 1.00, which resulted in a p-value of 0.006. Here the p-value indicates the proportion of subsequent trials (using the same design, intervention, and types of patients) which would result in an HR of 0.85 or more extreme, assuming the true *population* HR is 1. By convention, a p-value smaller than 0.05 is considered “significant”, however more stringent or liberal choices may also be applied. Aside from whether the CI rejects an HR of 1, the size of the 95%CI provides an indication of the variability of the HR, which in this case supports a smaller (HR of 0.96) or larger (HR of 0.76) benefit. Irrespective of the effect magnitude, there is reason to question the null-hypothesis HR of 1, where the data provides most support for an HR of 0.85.

In the aforementioned example, there is substantial evidence against the null-hypothesis, and hence, inference is fairly straightforward and uncontested. Conversely, interpreting results of “non-significant” analyses, where the null-hypothesis cannot be rejected, may leads to erroneous conclusions such as claiming a lack of “statistical significance” supports the null-hypothesis. For example, based on an HR of 0.86 (95%CI 0.40;1.87; p-value=0.71), the VOYAGER-PAD authors concluded that for the subgroup of patients with endovascular PAD, there was “no increase in intracranial or fatal bleeding”. ^5^ While it is clear that the null-hypothesis of no difference cannot be rejected, with the CI including an HR of 1.87, it is also clear that there is little evidence to support the absence of a harmful effect. Instead of claiming an absence of a risk-increasing effect, the presented results suggest that additional research is needed before drawing conclusions on bleeding risks.

In the following, we will discuss three common mistakes when interpreting results from statistical tests that fail to reject the null-hypothesis: 1) claiming that the null-hypothesis is true, 2) claiming that the study was underpowered, and 3) using multiple testing corrections to support claims about true or false associations.

## Why a non-significant result does not rule out a potentially meaningful association

There are two types of hypotheses: a strict null-hypothesis, where a supposed population parameter μ (e.g., the mean, mean difference, or hazard ratio) takes on a single value (e.g., $\mu_{0}=0$ ), and a composite null-hypothesis, where μ follows a range of values (e.g., $0.9<\mu_{0}<1.1$ ).

As with the illustrative examples, most often a strict null-hypothesis is (implicitly) evaluated. Given that a strict null-hypothesis postulates that μ is equal to a single value, it can be readily demonstrated that a statistical test cannot support the strict null-hypothesis. This understanding stems directly from the fact that for an arbitrary function ∫ its integral sum with limits a=b is zero (see Extended data for a formal proof ). More intuitively, to prove that μ is equal to any single value, one must obtain an estimate with zero bias and infinite precision. Essentially, this requires divine knowledge about the value of μ .

**Figure 1.**
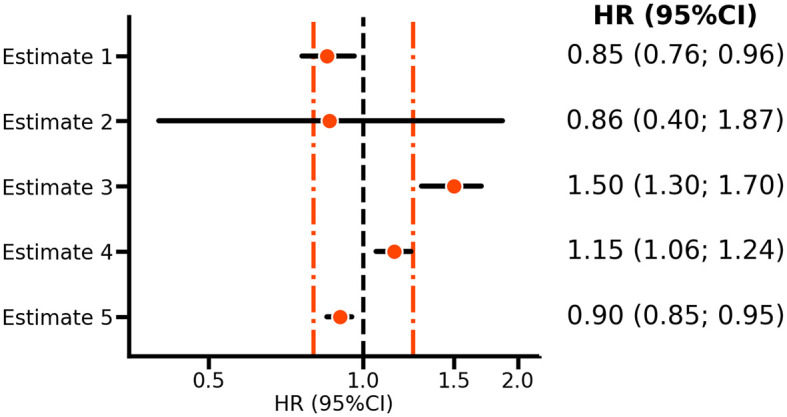
A forest plot illustrating non-inferiority and equivalence testing. N.b. Points represent hazard ratios (HR) with horizontal lines indicating 95% confidence intervals (CI). The orange vertical line indicates margins of equivalence at 0.80 and 1.25, with the vertical line at 1.00, indicating no difference. Estimates 1-2 are based on the VOYAGER PAD ^5^ rivaroxaban results for ischemic cardiovascular disease and bleeding risk, respectively. Estimates 3-5 are purely hypothetical and included as illustrations.

While a strict null-hypothesis cannot be supported by the data, no matter how much data is collected, there may often be a need to rule out certain effect(s). For example, the risk of bleeding when treating patients with rivaroxaban, or identifying interventions with limited efficacy. The solution is to simply use composite null-hypotheses. Returning to the illustrative example, one might consider a risk-increasing effect of 1.25 or less as sufficiently modest to not offset the observed benefit on ischemic cardiovascular disease. In this case, the null-hypothesis would be $\mu_{0}\geq 1.25$ , which would be rejected when both the estimated HR and the upper bound of the CI are smaller than 1.25 (or similarly using a one-sided test). For example, *estimate 2* in Figure 1 reflects the VOYAGER PAD estimate of the rivaroxaban bleeding effect, and it is clear that the confidence interval includes HRs above 1.25; hence, the null-hypothesis should not be rejected and there is no reason to conclude that rivaroxaban has a relatively small effect on bleeding risk. However, *estimate 4* is HR 1.15 (95%CI 1.06; 1.24) does exclude a HR larger than 1.25. Thus while *estimate 4* supports a risk increasing effect, one can nevertheless conclude that the effect size is sufficiently small to suggest that rivaroxaban is relatively safe.

This procedure is referred to as non-inferiority testing, where 1.25 is the bound of equivalence. Depending on what is deemed clinically non-inferior, such a bound can be substantially larger or smaller. For example, in the EBBINGHAUS trial of the PCSK9-inhibitor evolocumab the equivalence bound was set equal to 20% of the standard deviation of the cognitive function score measured in the placebo group. ^6^ The key characteristic of non-inferiority testing for safety is that both the point estimate (here, the HR) and the upper bound of the CI should be smaller than the supposed bound of equivalence. Given that one is not testing against a strict null-hypothesis of no difference, whether confidence (or statistical test) refutes a neutral value (e.g. an HR of 1) is immaterial. The consideration of two bounds, one on each side of a neutral HR of 1, naturally leads to equivalence testing. For example, in Figure 1, both *estimates 4* and *5* are deemed equivalent, despite both rejecting an HR of 1.0.

Defining bounds of equivalence or non-inferiority is challenging and a possible source of contention. Typically, such bounds are defined by combining statistical and clinical considerations. For example, evidence from previous studies can be meta-analysed to obtain a pooled effect estimate and confidence interval, where the confidence interval limits can be multiplied by a constants representing the amount of effect that one would like to preserve or rule out (for safety). ^7^

**Figure 2.**
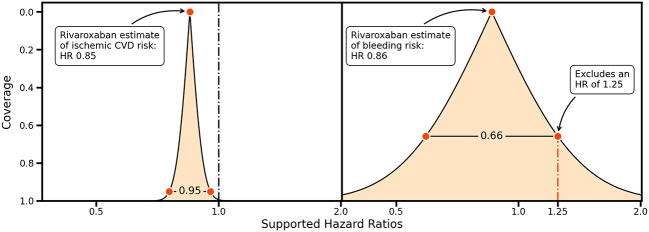
A compatibility graph comparing the confidence interval coverage against a range of hazard ratios for the rivaroxaban estimates on ischemic cardiovascular disease (left) and bleeding outcomes (right). Vertical lines indicate a hazard ratio (HR) of 1.00, and a possible margin of equivalence for an HR of 1.25. The shaded area indicates the HRs supported for a given coverage probability, indicated on the y-axis.

Ideas about non-inferiority and equivalence can be further generalized by considering the entire range of CIs providing information on precision and the HRs supported by collected data ^2^ ( Figure 2). For example, the rivaroxaban trial data support a wide range of bleeding risk HRs, and an HR of 1.25 would only be excluded using a 66%CI ( Figure 2, left panel). This can be contrasted by the small range of HRs supported by the rivaroxaban trial data for a protective effect on ischemic cardiovascular disease, indicating that the trial data are highly supportive of a protective effect ( Figure 2, right panel).

## Why post-hoc power provides the same information as a p-values and null-hypothesis test

Failure to reject a null-hypothesis naturally raises concerns about whether the study was sufficiently powered to detect a difference, often tempting researchers to conduct “post-hoc” or “observed” power calculations utilizing the observed point estimates (e.g. HRs) and variance estimates. Briefly, power reflects the probability of rejecting the null-hypothesis if it is false. This is the direct opposite of statistical tests and related quantities, such as p-values, which reflect the rejection probability assuming the null-hypothesis is true.

**Figure 3.**
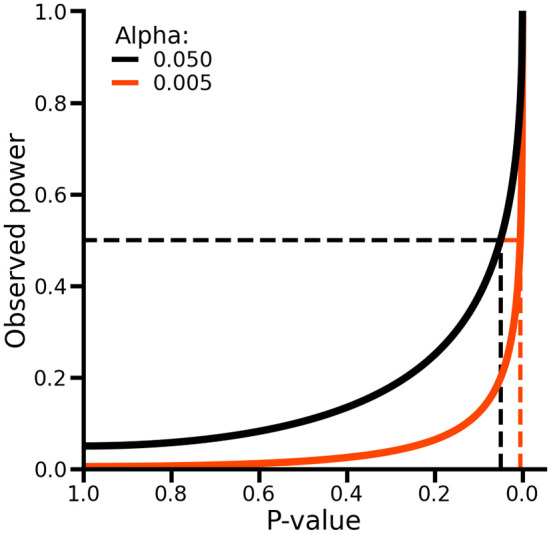
The relationship between p-values and observed power. Alpha refers to the type 1 error rate of a test. The dashed lines on the x-axis indicate the locations where the p-value is exactly equal to the alpha. The dashed lines on the y-axis indicate an observed power of 50%.

As such, p-values and observed power are equivalent, and no additional information is obtained by considering both ( Figure 3, see Extended data for a formal proof). To see this, suppose we observe a p-value of 0.05, which is equal to the conventional level of significance indicated by an alpha (i.e., the type 1 error rate) of 0.05. In this case, observed power can be obtained by calculating as probability of rejecting the null-hypothesis assuming the point estimates and it standard error are the true population values (which implies that the null-hypothesis is false), which in this case would be exactly 50% ( Figure 3). As such, while p-values evaluate the difference between the estimated values and the null-hypothesis assuming the former is true, power evaluates the same estimated values assuming the null-hypothesis is false. Hence, when p-values have been calculated, calculating observed power offers no additional information about the absence or presence of a difference.

More concerning is that post-hoc power calculations may lead to erroneous conclusions regarding the lack of statistical power or the absence of an effect. For example, the following two HR estimates both have a p-value of 0.71 and a power of 7%: HR 0.86 (95% CI 0.39; 1.91), HR 1.00 (95%CI 0.99; 1.01). Looking at the post-hoc power, one might conclude that both analyses were underpowered; however, the HR of 0.86 is non-significant due to considerable variability, whereas the latter HR of 1.00 simply reflects a clinically irrelevant association. Hence, a more relevant alternative to post-hoc power calculations is to evaluate the extent to which the CI includes clinically relevant effect estimates, which is in line with the aforementioned equivalence/non-inferiority approach. For example, the VOYAGER PAD HR estimate of 0.86 (95%CI 0.40;1.87) for bleeding risk in people with endovascular PAD clearly shows that the collected data supports a wide range of effect estimates, including potentially harmful associations. However, because the confidence interval only partially overlap with the proposed (hypothetical) upper bounds of acceptable harm of 1.25, testing against this bound results a p-value of 0.17 which is considerably smaller than testing against the complete absence of an effect: p-value 0.70. By comparison, the observed power estimate for these results is 7%, which implies that *if* the true HR was 0.86 one would have rejected the strict null-hypothesis in 7 out of 100 repeated experiment. As such observed power provide limit information relative to the presented alternative approaches, particularly the confidence interval based approach which allows for an informative discussion of benefits and harms in terms of effect magnitude(s).

Researchers may alternatively wish to calculate the power to reject a clinically meaningful difference other than the point estimate. Such calculations can meaningfully inform the design and viability of future studies; although sample size estimates may be more readily interpretable. However, when such power calculations are used to make statements about the presence or absence of an effect, or even lack of sample size, the described approach utilising confidence intervals and equivalence/non-inferiority margins provides more relevant information on study accuracy.

The futility of using power to make claims of the absence of an effect is further illustrated by noting that in the absence of an effect (i.e. when the p-value is 1) observed power is equal to the employed alpha threshold (e.g. 0.05). Hence, rather counterintuitively, low power may actually argue for the absence of an effect. Because power can only be calculated assuming the null-hypothesis is false, this metric cannot be used to make claims in favour of the null-hypothesis. Furthermore, as discussed in the preceding section, statistical tests cannot be used to support the strict null-hypothesis, as such this also holds for derived metrics such as p-value and power. While power remains essential when designing a future study, it should not be used to interpret results of a completed study. At this stage more relevant metrics such as confidence intervals are available which do not condition on the presence or absence of an effect, and provide information on accuracy as well as on effect magnitude.

## Why multiple testing correction does not differentiate between true and false results

Considerations of power and type 1 error rate are extremely relevant when designing a study, ensuring that a sufficiently accurate effect estimate may be realistically obtained given the available resources. However, both power and type 1 error rate are conditional probabilities assuming that the null-hypothesis is either true or false. As such these concepts are less relevant after the data have been collected, which would generally not consist of null-hypothesises which are either all true or all false, but instead will include an unknown mixture of both. It is important to realize that the type 1 error rate itself does not reflect the proportion of false positive results, but merely reflects the expected proportion of false positive results should *all null-hypotheses be true.*

**Figure 4.**
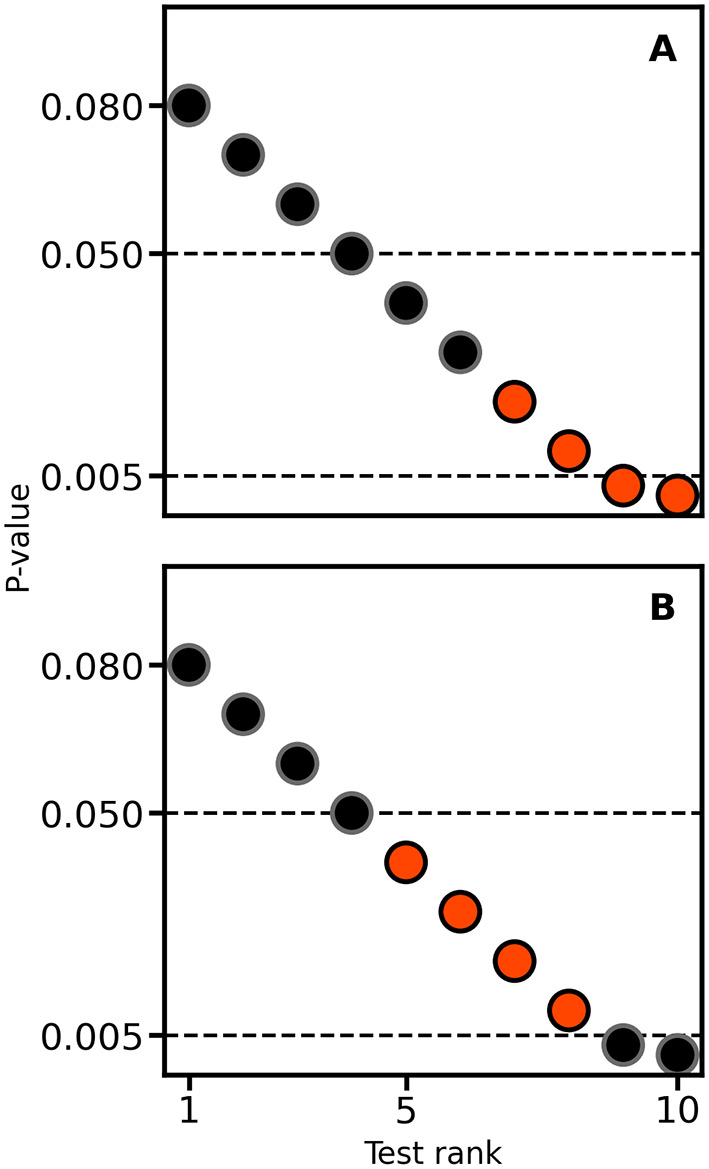
Illustrating the impact of multiple testing correction on the false discovery rate. In each panel, four tests are true positives (orangered) and six are false positives (black). Horizontal lines are drawn at 0.05 and 0.005, the latter reflecting a Bonferroni correction for the 10 applied null-hypothesis tests.

When considering the results of two or more null-hypothesis tests, in an attempt to decrease the number of false positive results, there is an expectation to perform multiple testing corrections. For example, the Bonferroni method is a popular multiplicity correction evaluating p-values against an alpha (e.g., 0.05) divided by the number of conducted tests. It is well known that post-hoc multiple testing correction (e.g., corrections not accounted for during the design stage by increasing the collected sample size) will decrease power ( Figure 3). An often overlooked point is that, depending on the unknown balance between false positives and true positives in a set of test results, applying multiplicity correction can sometimes increase the false discovery rate (i.e., the fraction of false positives divided by the total number of positive tests) instead of reducing it. For example, Figure 4A presents a naive expectation of multiple testing corrections, where the false discovery rate decreases from 1/3 to 0. However, there is no reason why the scenario depicted in Figure 4B may not occur; here, the false discovery rate increases from 1/3 to 1.

**Figure 5.**
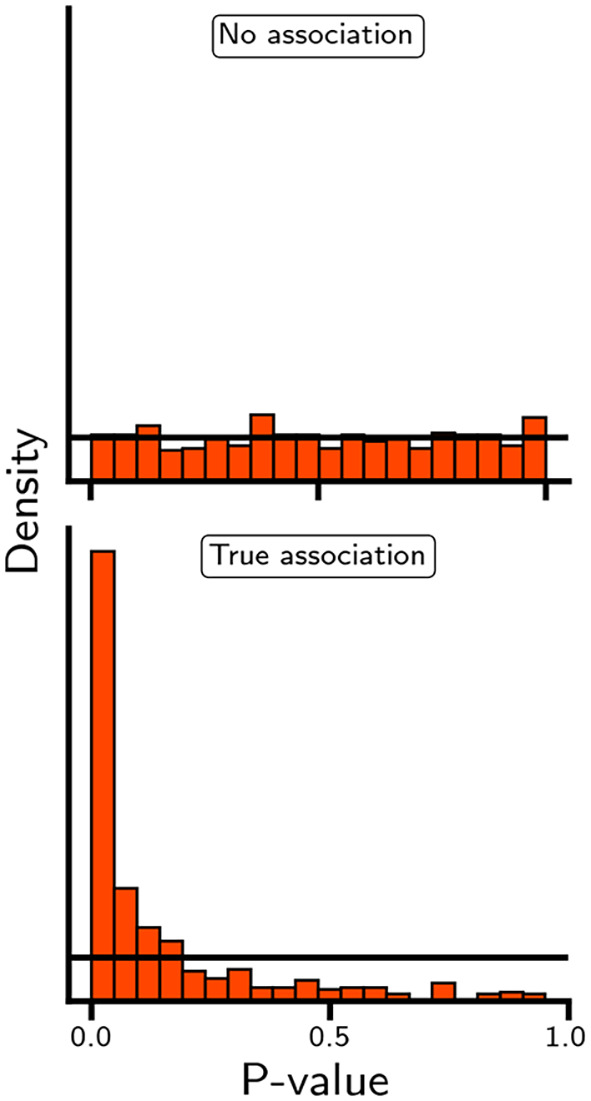
The distribution of p-values when the null-hypothesis is true or false. N.b. The p-values were derived by arbitrarily sampling 1,000 test statistic from a normal distribution and leveraging its cumulative density function to calculate the area on the left and right side of the sampled test-statistic. Specifically, the employed standard distribution had a standard deviation of 1 and mean of either 0 or 2, when the null-hypothesis was true and false, respectively. Please note that the normal distribution is only used as an exemplar, and alternative distributions with a known cumulative density function (e.g. chi-square, beta, or gamma) could have been used instead.

As shown in Figure 5, in which the distribution of p-values is generated in the absence and presence of an association, small p-values may occur in both settings. As such, while a small p-value (or equivalently, a extreme test statistic) is unlikely to occur when there is no association, observing a single small p-value is insufficient to differentiate between true and false positive results. Concepts such as indirect or direct replication and internal consistency are more relevant to differentiate between true and false positives. For example, in the case of rivaroxaban, associations with multiple types of ischemic cardiovascular events (e.g., myocardial infarction, ischemic stroke, and acute limb ischemia) will be more convincing than an association with any one outcome.

While individual p-values and null-hypothesis tests cannot differentiate between false and true positive results, a set of p-values ( Figure 5) can be compared against a uniform distribution to determine the likelihood that the entire set is driven by false positive results. This approach is independent of the specific statistical test used to derive individual p-values. Moreover, the method can be generalised to account for dependencies among p-values, such as dependencies arising from the inclusion of both composite and individual outcomes (e.g., evaluating both any stroke and ischaemic stroke). Similarly, one can determine the proportion of p-values that are smaller than a predefined alpha; for example, in Figure 5, the proportion of p-values smaller than 0.05 is of course 0.05 for the top panel and 0.49 for the lower panel. Returning to our illustrative example, despite showing a potentially protective association with myocardial infarction, ischemic stroke, major amputation, and venous thromboembolism, the null-hypothesis could only be rejected for the association between rivaroxaban and acute limb ischemia: HR 0.67 (95% 0.55; 0.82). ^4^ Utilizing a non-parametric Kolmogorov-Smirnov test to compare the set of p-values for all the aforementioned outcomes against a uniform distribution nevertheless resulted in a p-value of 0.02, suggesting that the protective effect of rivaroxaban is shared across multiple cardiovascular outcomes.

## Discussion

In the current manuscript, we have addressed why statistical tests cannot be used to support the strict null-hypothesis. Instead, concerns regarding the safety or lack of efficacy should be evaluated using equivalence testing. This can be readily implemented in any study by combining confidence intervals with bounds of clinical insignificance. Such an approach provides direct information on whether a non-significant test is due to an association and its variability being sufficiently small or simply reflects a lack of accuracy. Furthermore, contrary to expectation and depending on the unknown proportion of true positive results, multiple testing corrections may increase the false discovery rate. Finally, because power and type 1 error rate make extreme assumptions about whether all results are true or false positives, these concepts have limited relevance after the data have been collected and analysed.

The current manuscript provides guidance on how standard null-hypothesis testing can be used to provide clinically meaningful insights, and attempts to move beyond the current erroneous modus vivendi, categorizing associations as true and false. Contrary to recent calls to completely abandon significance testing, ^2^ this contribution calls for a more considerate and bespoke application of the currently available and ubiquitously accepted methods. Specifically, researchers should routinely indicate bounds between which an effect is sufficiently small to be considered clinically irrelevant. Related to this, the idea that any intervention should (or even can) be without harmful side effects needs to be dismissed and replaced with a notion of benefit versus harm, where clinically supported bounds off irrelevance can help to directly inform. Second, while notions of power and type 1 errors are essential at the study design phase, because these deal in hypothetical scenarios where all results are either true or false such metrics have limited relevance when interpreting results. Power and type 1 errors can be framed in terms of probabilities *because* the analysis has not yet been conducted. Once the experiment has been completed, these hypothetical probabilities are immaterial, and one is simply confronted with an unknown proportion of true-positive results. At this stage, concepts of power and type 1 error must be replaced by indicators of precision, such as confidence intervals. Instead of using confidence intervals as a proxy for null-hypothesis testing (i.e., whether the null-hypothesis value is excluded), inference should focus on determining too what extent there is sufficient precision to exclude meaningful differences. Finally, while decreasing the significance threshold (e.g. from 0.05 to 0.005) decreases the type 1 error rate this decreases power as well, and hence may decrease the number of true associations discovered. Depending on the area of research overlooked, true positive results may be more harmful than false positive results. For example, protein drug targets identified in early drug development are often subjected to a substantial number of follow-up analyses, which filter out false positive results. Such follow-up studies, however, rarely expand the number of candidates, hence suggesting a more inclusive approach might be more considerate. In settings more proximal to clinical implementation and less discovery oriented, such as phase 3 clinical trials, stringent multiple testing correction is clearly called for. Notwithstanding, it is important to realize that not every study needs to be designed as a clinical trial.

In conclusion, failure to reject a strict null-hypothesis does not support the absence of a clinically meaningful association. Instead, researchers should routinely apply composite null-hypothesis tests evaluated against meaningful bounds of insignificance. Genuine consideration of estimation accuracy, as provided through confidence intervals, precludes the need for questionable post-hoc power calculations. Finally, because power and type 1 errors unrealistically assume that all results are true or false, these concepts have limited value once data collection and analysis have been completed.

## Author contributions

AFS designed the illustrations and wrote the manuscript.

## Ethics and consent

Ethics and consent were not required.

## Data Availability

No data associated with this article. Addressing common inferential mistakes when failing to reject the null-hypothesis https://doi.org/10.5522/04/27854043. ^8^ Data are available under the terms of the Creative Commons Attribution 4.0 International license (CC-BY 4.0).
