# Supplementary material for: Addressing common inferential mistakes when failing to reject the null-hypothesis
Source: F1000Res. 2025 Apr 1;13:1488. Originally published 2024 Dec 5. [Version 3] doi: 10.12688/f1000research.158434.3 (PMC11928781; doi:10.12688/f1000research.158434.3)
Supplement: Peer review history

# Reviewer report: Reviewer response for version 2

**DOI:** 10.5256/f1000research.177945.r368387

**Published:** 2025-03-21

**Authors:** J. Alexander Heimel

**Affiliations:** 1 Netherlands Institute for Neuroscience, Amsterdam, The Netherlands

The author has answered my comments to my satisfaction, except for one small point.

 In his reply to my question, the author wrote that the relationship between p-value and observed power is independent of sample size n. I do not believe this to be true. In the new supplementary material, the author sketches this relationship, but ignores the fact the t-distribution (denoted as g in the supplementary material) is dependent on the degrees of freedom (d.o.f. = n-1) and therefore on the sample size. The value t_c will thus depend on n and the observed power, given by the author as g(t_0-t_c)+g(-t_0-t_c) will also depend on n, through g and t_c. In fact, I believe the correct formula for observed power should be:

 Observed_power(t) = g_{d.o.f, t}(t_c) + g_{d.o.f, -t}(t_c)

 With g_{d.o.f.,t}(t_c) the cumulative non-central t-distribution with d.o.f. degrees of freedom shift t evaluated at t_c.  

 For large n, the approximations used by the author converge, and the dependence on the sample size disappears.

 This dependence on sample size, however, does not fundamentally change or invalidate the author’s argument that a post-hoc power calculation using the observed effect is not useful.

 While studying the relationship between observed power and p-value for my reply, I came across the Wikipedia page on power analysis that also makes the author’s point that post-hoc power analysis is fundamentally flawed, with references to Hoenig (The American Statistician, 2001), cited more than 2000 times, and Thomas (Conservation Biology, 1997) already pointing out the same flaw. The author’s case is thus not new, but may still be very relevant and a good reminder. It certainly was for me.

 Minor details about the new supplementary note:

 I think that when writing “the p-value for a one-sided tests would simply be 1-g(|t_0|)”, already the assumption of f being symmetrical about zero is made. The statement is not true for a general f and t_0<0.

 “In this case the two-sided p-value is 2x(1-g(2)) \approx 0.46”. I believe this should be “\approx 0.046” (and is only true for large sample size).

Is the topic of the opinion article discussed accurately in the context of the current literature?

Partly

Are arguments sufficiently supported by evidence from the published literature?

Partly

Are all factual statements correct and adequately supported by citations?

Partly

Are the conclusions drawn balanced and justified on the basis of the presented arguments?

Partly

Reviewer Expertise:

Neuroscience

I confirm that I have read this submission and believe that I have an appropriate level of expertise to confirm that it is of an acceptable scientific standard.

## References

[1] : The Abuse of Power. *The American Statistician* .2001;55(1) : 10.1198/000313001300339897 19-24 10.1198/000313001300339897

[2] Thomas, Len. "Retrospective power analysis." Conservation Biology 11.1 (1997): 276-280.

# Response

**Published:** 2025-03-25

**Authors:** Amand Schmidt

**Affiliations:** University College London Faculty of Population Health Sciences, London, England, UK

Reviewer 2

**Comment: **The author has answered my comments to my satisfaction, except for one small point.

 In his reply to my question, the author wrote that the relationship between p-value and observed power is independent of sample size n. I do not believe this to be true. In the new supplementary material, the author sketches this relationship, but ignores the fact the t-distribution (denoted as g in the supplementary material) is dependent on the degrees of freedom (d.o.f. = n-1) and therefore on the sample size. The value t_c will thus depend on n and the observed power, given by the author as g(t_0-t_c)+g(-t_0-t_c) will also depend on n, through g and t_c. In fact, I believe the correct formula for observed power should be:

 Observed_power(t) = g_{d.o.f, t}(t_c) + g_{d.o.f, -t}(t_c)

 With g_{d.o.f.,t}(t_c) the cumulative non-central t-distribution with d.o.f. degrees of freedom shift t evaluated at t_c. 

 For large n, the approximations used by the author converge, and the dependence on the sample size disappears.

 This dependence on sample size, however, does not fundamentally change or invalidate the author’s argument that a post-hoc power calculation using the observed effect is not useful.

**Response: **I am pleased to note that the reviewer agrees with the main conclusions of the manuscript.****

 The reviewer appears to be referring to the sample size–dependent convergence of the *t*-distribution to the standard normal distribution. While this is, of course, entirely correct and uncontested, it is not directly relevant to the point under discussion. The relationship between p-values and statistical power is independent of the choice of distribution, and does not rely on any asymptotic approximation.

 Although the illustrative examples in the manuscript use the standard normal distribution -which does not depend on sample size or degrees of freedom - the relationship between p-value and power holds equally when using distributions that do, such as the t- or F-distributions. This is because both the *p*-value and power are calculated using the same distribution, with the same degrees of freedom. For example, if one calculates a p-value using a t-distribution with 7 degrees of freedom, the corresponding power would also be calculated using that *same t-distribution* with 7 degrees of freedom. Thus, while the distribution itself may depend on sample size, the relationship between p-value and power remains exact and independent of sample size.

 The following was added to the supplementary to help clarify this.

 “

*As in the previous section, these expressions do not depend on the amount of data collected and are therefore exact. If one is uses a statistical distribution with degrees of freedom (which often depend on sample size), such as the t or F distributions, the relationship between the p-value and power remain exact, simply because both quantities are calculated using the same distribution with the same degrees of freedom.*

 “

**Comment: **While studying the relationship between observed power and p-value for my reply, I came across the Wikipedia page on power analysis that also makes the author’s point that post-hoc power analysis is fundamentally flawed, with references to Hoenig (The American Statistician, 2001), cited more than 2000 times, and Thomas (Conservation Biology, 1997) already pointing out the same flaw. The author’s case is thus not new, but may still be very relevant and a good reminder. It certainly was for me.

**Response: **Thank you these reference have been included in the updated manuscript.

**Comment: **Minor details about the new supplementary note:

 I think that when writing “the p-value for a one-sided tests would simply be 1-g(|t_0|)”, already the assumption of f being symmetrical about zero is made. The statement is not true for a general f and t_0<0.

**Response: **This section briefly describes p-values for one-sided and two-sided tests.

 The section has been clarified to explain that this statement is not an assumption but merely reflects a known quantity about some distributions being symmetrical about zero where others are not. The expanded section now provides examples of alternative distributions which are not symmetrical about zero. The reason that this was not originally included is that commonly known non-symmetrical distribution such as the Chi-square, the F distribution, or even the Gamma distribution are not defined for negative values making them less applicable for two-sided tests.

 “

*Next, say we observe test-statistic* *t* *o*  *, in that case the p-value for a one-sided tests would simply be* *1-g* *t* *o* *,*  *and assuming* *f*  *is symmetrical about zero (e.g. the normal distribution or t-distribution) the p-value for a two-sided test would simply be* *2×* *1-g* *t* *o*  *. For distributions that are not symmetric about zero, both sides of the distribution should be considered separately. It is important to note, however, that many commonly used asymmetric distributions—such as the F, Chi-squared, and Gamma distributions—are defined only for positive values. This restricts their direct applicability for two-sided hypothesis testing.*

 “

**Comment: **“In this case the two-sided p-value is 2x(1-g(2)) \approx 0.46”. I believe this should be “\approx 0.046” (and is only true for large sample size).

**Response: **Thanks for spotting this type-o, this should be 0.046 indeed.

 However, this is not dependent on any sample size assumption. The approximate symbol (≈) is used purely to reflect rounding to three decimal places, not to imply asymptotic behaviour or large-sample properties.

 It’s possible the reviewer is conflating this with the well-known approximation of the *t*-distribution by the standard normal distribution as sample size increases. While that is certainly valid in a different context, it is not relevant here. I am not making any claims about inference or distributional convergence; rather, I am simply referring to the numerical value of the cumulative distribution function.

 To clarify: if *g *denotes the cumulative distribution function of the standard normal distribution, then *2×(1-g* *2* *≈0.046*, regardless of sample size. Similarly, if one were to use the *t*-distribution with 7 degrees of freedom, the corresponding value would be approximately 0.086 - the choice of 7 here being arbitrary and illustrative.

 This is a purely mathematical statement about evaluating a distribution function at a specific point, and not a comment on sampling or estimation.

# Reviewer report: Reviewer response for version 2

**DOI:** 10.5256/f1000research.177945.r368388

**Published:** 2025-03-06

**Authors:** Ying Cui

**Affiliations:** 1 Stanford University, Stanford,, California, USA

No further comments.

Is the topic of the opinion article discussed accurately in the context of the current literature?

Yes

Are arguments sufficiently supported by evidence from the published literature?

Yes

Are all factual statements correct and adequately supported by citations?

Yes

Are the conclusions drawn balanced and justified on the basis of the presented arguments?

Yes

Reviewer Expertise:

Hypothesis testing, Biostatistics

I confirm that I have read this submission and believe that I have an appropriate level of expertise to confirm that it is of an acceptable scientific standard.

# Reviewer report: Reviewer response for version 1

**DOI:** 10.5256/f1000research.174018.r350494

**Published:** 2025-01-07

**Authors:** J. Alexander Heimel

**Affiliations:** 1 Netherlands Institute for Neuroscience, Amsterdam, The Netherlands

The manuscript, “Addressing common inferential mistakes when failing to reject the null-hypothesis” contains two main messages. First, the author argues that post-hoc power calculations should be avoided, as “they offer no additional information beyond statistical tests and p-values” and they “can be misleading because of an inability to distinguish between results based on insufficient sample size and results that reflect clinically irrelevant differences”. Second, for interpreting the results of multiple comparison, the authors recommends to consider “the distribution of p-values and the proportion of significant results to identify bodies” rather than standard multiple testing procedures, because they “unrealistically assume that all positive results are false positive”. The paper is an interesting read, but I have a few remarks.

 I have two comments on the first point.

 “p-values and observed power are equivalent, and no additional information is obtained by considering both ( Figure 3).”  No proof is given of this statement and it is not completely trivial. Can a (hint of a) proof be given or a reference with a proof cited? I believe the relationship shown and figure 3 between p and power depends on the number of samples, but this is not shown in the text or figure. Can you comment on this? The dependence on the sample number could give some added value for making this post-hoc power calculation, and p-values and observed power are thus not completely equivalent.

 Are there examples that people do this particular post-hoc power calculation using the measured effect and the measured standard deviation? What I have encountered, is post-hoc power calculations based on the measured standard deviation of the sample, and for an assumed effect size (rather than the measured effect). If there are publications doing the post-hoc power analysis in the way suggested by this publication, then please give some example references. Otherwise, the reader is left to wonder if this part of the manuscript is arguing against hypothetical reasoning that people do not actually use.

 The second point of the paper is that “While individual p-values and null hypothesis tests cannot differentiate between false and true positive results, a set of p-values (Figure 5) can be compared against a uniform distribution to determine the likelihood that the entire set is driven by false positive results.” This can indeed be helpful. However, if assumptions like independence between tests or normality of the measurements for some tests fail, then this meta-analysis on the p-value may give a false positive outcome. Without a good understanding of the underlying data and statistics, I would use this test only to suggest that results are possibly false positives if the distribution of p-values is not distinguishable from a uniform distribution, rather than concluding that there must be some true positive effect if the distribution of p-values is not uniform, as is done by the author for the specific example for the effect of rivaroxaban.

 These are my main comments. I also have a number of smaller remarks.

 The manuscript also discusses whether null hypothesis testing can prove whether difference in means between two (infinite) populations is truly zero, but I fail to see the connection of this more philosophical point on the interpretation of hypothesis testing to the main messages. I think that the manuscript would be stronger if it is left out.

 The meaning of the sentence “Given that power and type 1 error make extreme assumptions where either all results are true or false positives, these concepts are less relevant after the data have been collected. ” is not at all clear to me. Perhaps rephrase to clarify.

 “applying multiplicity correction can sometimes increase the false positive rate instead of reducing it.” In this sentence, and the ensuing section the term “false positive rate” is used differently from what is the common use in the statistical literature. In the literature, “false positive rate” is the expectation of a false positive result when repeating the procedure. The meaning that is taken in this manuscript is that of the fraction of the significant results of a given set of experiments that is a false positive. It is not clear that the interpretation chosen here, and which is shown by example to lead to a false expectation, actually often occurs in the scientific literature. Can some example references be provided?

 Fig. 5: The number of samples used for producing the figure is missing.

 “comparing the set of p-values for all the aforementioned outcomes against a uniform distribution resulted in a p-value of 0.02,” Which test is used for the comparison.

 “researchers should routinely indicate the bounds between which an effect is sufficiently small to be considered clinically irrelevant” This is a valuable recommendation. Before the start of a study it is necessary to choose an expected effect size for determining the power of a study or the number of samples. If the expected effect size, is below the bound, then either the study should not be started (if one is looking to prove a positive effect) or the bound should be taken as the effect size for the power calculation.

 “Second, while notions of power and type 1 errors are essential at the study design phase because these deal in hypothetical scenarios where all results are either true or false, such metrics have limited relevance when interpreting results.” I do not fully agree. For a power analysis at the design phase, typically both an effect size and the variance need to be estimated. In my opinion, a post-hoc power analysis could be relevant for interpreting a negative result, to take into account the observed variance. If the variance is a lot larger than a priori expected, then the study could have been underpowered. Perhaps adjust the text, or explain why I am wrong.

 I do not understand “Finally, depending on the area of research overlooked, true positive results may be more harmful than false positive results”. Is meant that setting too stringent boundaries for significance can be harmful? Consider rephrasing to make its meaning clear.

Is the topic of the opinion article discussed accurately in the context of the current literature?

Partly

Are arguments sufficiently supported by evidence from the published literature?

Partly

Are all factual statements correct and adequately supported by citations?

Partly

Are the conclusions drawn balanced and justified on the basis of the presented arguments?

Partly

Reviewer Expertise:

Neuroscience

I confirm that I have read this submission and believe that I have an appropriate level of expertise to confirm that it is of an acceptable scientific standard, however I have significant reservations, as outlined above.

# Response

**Published:** 2025-02-04

**Authors:** Amand Schmidt

**Affiliations:** University College London Faculty of Population Health Sciences, London, England, UK

**The manuscript, “Addressing common inferential mistakes when failing to reject the null-hypothesis” contains two main messages. First, the author argues that post-hoc power calculations should be avoided, as “they offer no additional information beyond statistical tests and p-values” and they “can be misleading because of an inability to distinguish between results based on insufficient sample size and results that reflect clinically irrelevant differences”. Second, for interpreting the results of multiple comparison, the authors recommends to consider “the distribution of p-values and the proportion of significant results to identify bodies” rather than standard multiple testing procedures, because they “unrealistically assume that all positive results are false positive”. The paper is an interesting read, but I have a few remarks.**

**I have two comments on the first point.**

**“p-values and observed power are equivalent, and no additional information is obtained by considering both ( Figure 3).”  No proof is given of this statement and it is not completely trivial. Can a (hint of a) proof be given or a reference with a proof cited? I believe the relationship shown and figure 3 between p and power depends on the number of samples, but this is not shown in the text or figure. Can you comment on this? The dependence on the sample number could give some added value for making this post-hoc power calculation, and p-values and observed power are thus not completely equivalent.**

**Response: **thank you, the following proof is provided in the updated supplementary material, also confirming this relationship is independent of sample size.

 “

*To describe the relationship between p-values and observed power we first let* *g*  *represent the commulative density function of the p.d.f.* *f*  *, that is*

*g* *t* *=* *-∞* *t* *f* *x* *dx* *.* 

*Next, say we observe test-statistic* *t* *o*  *, in that case the p-value for a one-sided tests would simply be* *1-g* *t* *o* *,*  *and assuming* *f*  *is symmetrical about zero the p-value for a two-sided test would simply be* *2×* *1-g* *t* *o*  *.*

*If we let* *t* *c*  *represent the critical value of a statistical test beyond which the null-hypothesis is rejected, power can be calculated as* *g* *t* *o* *-tc* *+g(* *-t* *0* *-* *t* *c* *)*  *, where in most cases either the first or the second term will be close to zero and can be ignored.*

*As an example, let* *f*  *be the p.d.f. of the standard normal distribution, with* *t* *0* *=2*  *and* *t* *c* *=1.96*  *. In this case the two-sided p-value is* *2×* *1-g* *2* *≈0.46*  *, and power would be* *g* *2-1.96* *+g* *-2-1.96* *≈0.52+0.00=0.52.*  *Similarly, say* *t* *0* *=0*  *(i.e., the null-hypothesis is true), and* *f*  *and* *t* *c*  *are the same as before, we then find that* *2×* *1-g* *0* *=1*  *and* *g* *-1.96* *+g* *-1.96* *=0.05*  *. In other words when the null-hypothesis is true the p-value would be 1 and power would be 0.05 (or more generally power would equal to the area excluded by* *t* *c*  *).*

*As such there exists a one-to-one relationship between the p-value and observed power based on* *t* *o*  *. Furthermore, and perhaps counterintuitively observed power is smallest when the p-value provides most support for the null-hypothesis and vice versa. This is a reflection of the p-value evaluating* *t* *o*  *assuming the null-hypothesis is true, whereas observed power evaluates* *t* *o*  *assuming this hypothesis is false. As in the previous section, these expressions do not depend on the amount of data collected and are therefore exact. Furthermore, given that a statistical test, comparing* *t* *o*  *to* *t* *c*  *, cannot be used to prove the null-hypothesis is true (as shown in the preceding section), the same holds for derived metrics including p-values and power. In fact power can only be calculated assuming the null-hypothesis is false, and hence cannot be used to argue in favour of the null-hypothesis being true.*

 “

**Are there examples that people do this particular post-hoc power calculation using the measured effect and the measured standard deviation? What I have encountered, is post-hoc power calculations based on the measured standard deviation of the sample, and for an assumed effect size (rather than the measured effect). If there are publications doing the post-hoc power analysis in the way suggested by this publication, then please give some example references. Otherwise, the reader is left to wonder if this part of the manuscript is arguing against hypothetical reasoning that people do not actually use.**

**Response: **Thank you for raising this important point. The manuscript has been updated to clarify that the raised issues with observed power are of general concern irrespective of the whether the point estimates reflect the study results or some pre-existing notion of relevant difference. The concerns about power pertain to its use as a metric to interpret *current *results. In doing so the manuscript more clearly differentiates with power and/or sample size calculations to inform future research (which are without fault) and power calculations for already completed studies.

 Page 6-7

*“*

*Researchers may alternatively wish to calculate the power to reject a clinically meaningful difference other than the point estimate. Such calculations can meaningfully inform the design and viability of future studies; although sample size estimates may be more readily interpretable. However, when such power calculations are used to make statements about the presence or absence of an effect, or even lack of sample size, the described approach utilising confidence intervals and equivalence/non-inferiority margins provides more relevant information on study accuracy.*

*The futility of using power to make claims of the absence of an effect is further illustrated by noting that in the absence of an effect (i.e. when the p-value is 1) observed power is equal to the employed alpha threshold (e.g. 0.05). Hence, rather counterintuitively, low power may actually argue for the absence of an effect. Because power can only be calculated assuming the null-hypothesis is false, this metric cannot be used to make claims in favour of the null-hypothesis. Furthermore, as discussed in the preceding section, statistical tests cannot be used to support the strict null-hypothesis, as such this also holds for derived metrics such as p-value and power. While power remains essential when designing a future study, it should not be used to interpret results of a completed study. At this stage more relevant metrics such as confidence intervals are available which do not condition on the presence or absence of an effect, and provide information on accuracy as well as on effect magnitude. “*

 As well as page 9

 “

*Second, while notions of power and type 1 errors are essential at the study design phase, because these deal in hypothetical scenarios where all results are either true or false,  such metrics have limited relevance when interpreting results. Power and type 1 errors can be framed in terms of probabilities because the analysis has not yet been conducted. Once the experiment has been completed, these hypothetical probabilities are immaterial, and one is simply confronted with an unknown proportion of true-positive results.*

 “

**The second point of the paper is that “While individual p-values and null hypothesis tests cannot differentiate between false and true positive results, a set of p-values (Figure 5) can be compared against a uniform distribution to determine the likelihood that the entire set is driven by false positive results.” This can indeed be helpful. However, if assumptions like independence between tests or normality of the measurements for some tests fail, then this meta-analysis on the p-value may give a false positive outcome. Without a good understanding of the underlying data and statistics, I would use this test only to suggest that results are possibly false positives if the distribution of p-values is not distinguishable from a uniform distribution, rather than concluding that there must be some true positive effect if the distribution of p-values is not uniform, as is done by the author for the specific example for the effect of rivaroxaban.**

**Response: **Thanks for raising these important points. The manuscript has been updated to clarify this involves a general testing procedure which does not make assumptions on the test statistics or the set of p-values following a normal distribution. Furthermore, the manuscript now explains that there may be a need to account for dependencies between sets of p-values.

 The following has been included on page 8 “

*While individual p-values and null-hypothesis tests cannot differentiate between false and true positive results, a set of p-values (* *Figure 5* *) can be compared against a uniform distribution to determine the likelihood that the entire set is driven by false positive results.* *This approach is independent of the specific statistical test used to derive individual p-values. Moreover, the method can be generalised to account for dependencies among p-values, such as dependencies arising from the inclusion of both composite and individual outcomes (e.g., evaluating both any stroke and ischaemic stroke).*

 “

 And the figure 5 note “

*N.b. The p-values were derived by arbitrarily sampling 1,000 test statistic from a normal distribution and leveraging its cumulative density function to calculate the area on the left and right side of the sampled test-statistic. Specifically, the employed  standard distribution had based on random draws from a normal distribution with a a standard deviation of 1 and mean of either 0 or 2, when the null-hypothesis was true and false, respectively. Please note that the normal distribution is only used as an exemplar, and alternative distributions with a known cumulative density function (e.g. chi-square, beta, or gamma) could have been used instead.*

 “

**These are my main comments. I also have a number of smaller remarks.**

**The manuscript also discusses whether null hypothesis testing can prove whether difference in means between two (infinite) populations is truly zero, but I fail to see the connection of this more philosophical point on the interpretation of hypothesis testing to the main messages. I think that the manuscript would be stronger if it is left out.**

**Response: **This has now been integrated with the sections describing power, to further support the argumentation that (observed) power calculations are futile because the null-hypothesis can never be empirically shown to be true. As such this section is far from philosophical or redundant.

**The meaning of the sentence “Given that power and type 1 error make extreme assumptions where either all results are true or false positives, these concepts are less relevant after the data have been collected. ” is not at all clear to me. Perhaps rephrase to clarify.**

**Response: **The following clarification was added.

 Page 7

 “

*Considerations of power and type 1 error rate are extremely relevant when designing a study, ensuring that a sufficiently accurate effect estimate may be realistically obtained given the available resources. However, both power and type 1 error rate are conditional probabilities assuming that the null-hypothesis is either true or false. As such these concepts are less relevant after the data have been collected, which would generally not consist of null-hypothesises which are either all true or all false, but instead will include an unknown mixture of both.*

 “

**“applying multiplicity correction can sometimes increase the false positive rate instead of reducing it.” In this sentence, and the ensuing section the term “false positive rate” is used differently from what is the common use in the statistical literature. In the literature, “false positive rate” is the expectation of a false positive result when repeating the procedure. The meaning that is taken in this manuscript is that of the fraction of the significant results of a given set of experiments that is a false positive. It is not clear that the interpretation chosen here, and which is shown by example to lead to a false expectation, actually often occurs in the scientific literature. Can some example references be provided?**

**Response: **Thanks for pointing this out, the manuscript has been updated to refer to this as the false discovery rate, including a formal definition.

 Page 7

 “

 An often overlooked point is that, depending on the unknown balance between false positives and true positives in a set of test results, applying multiplicity correction can sometimes increase the false discovery rate (i.e., the fraction of false positives divided by the total number of positive tests) instead of reducing it. For example, Figure 4A presents a naive expectation of multiple testing corrections, where the false discovery rate decreases from 1/3 to 0. However, there is no reason why the scenario depicted in Figure 4B may not occur; here, the false discovery rate increases from 1/3 to 1.

 “

 Given that the false discovery rate can only be defined based on the knowledge about which hypotheses are true and which are false, the second part of the question is impossible to answer for empirical research. However, the following thought experiment can provide some intuition. We know that the incidence of cardiovascular disease increases with age, which implies that in a sufficiently large study there would be a significant association between age and cardiovascular disease, say the p-value is 0.001. If we conduct a single test at an alpha (type 1 error rate) of 0.05 we would correctly reject a null hypothesis of no association. If we subsequently preformed four additional tests of association using completely random pairs of variables (i.e., where the null hypothesis is true), the Bonferroni multiplicity corrected alpha would be 0.01 and we would not reject the null hypothesis that age associates with cardiovascular disease. If however due to random chance one of the p-value for one of the remaining tests is smaller than 0.01 we would incorrectly reject the null-hypothesis thereby increasing the false discovery rate from 0 (when we only test for age) to 1/5.

**Fig. 5: The number of samples used for producing the figure is missing.**

**Response: **This was added.

**“comparing the set of p-values for all the aforementioned outcomes against a uniform distribution resulted in a p-value of 0.02,” Which test is used for the comparison.**

**Response: **This has been added.

 “

*Utilizing a non-parametric Kolmogorov-Smirnov test to compare the set of p-values for all the aforementioned outcomes against a uniform distribution nevertheless resulted in a p-value of 0.02, suggesting that the protective effect of rivaroxaban is shared across multiple cardiovascular outcomes.*

 “

**“researchers should routinely indicate the bounds between which an effect is sufficiently small to be considered clinically irrelevant” This is a valuable recommendation. Before the start of a study it is necessary to choose an expected effect size for determining the power of a study or the number of samples. If the expected effect size, is below the bound, then either the study should not be started (if one is looking to prove a positive effect) or the bound should be taken as the effect size for the power calculation.**

**Response: **Thank you.

**“Second, while notions of power and type 1 errors are essential at the study design phase because these deal in hypothetical scenarios where all results are either true or false, such metrics have limited relevance when interpreting results.” I do not fully agree. For a power analysis at the design phase, typically both an effect size and the variance need to be estimated. In my opinion, a post-hoc power analysis could be relevant for interpreting a negative result, to take into account the observed variance. If the variance is a lot larger than a priori expected, then the study could have been underpowered. Perhaps adjust the text, or explain why I am wrong.**

**Response: **The requested explanation has been included. Please refer to page 7-8.

 “

*Researchers may alternatively wish to calculate the power to reject a clinically meaningful difference other than the point estimate. Such calculations can meaningfully inform the design and viability of future studies; although sample size estimates may be more readily interpretable. However, when such power calculations are used to make statements about the presence or absence of an effect, or even lack of sample size, the described approach utilising confidence intervals and equivalence/non-inferiority margins provides more relevant information on study accuracy.*

*The futility of using power to make claims of the absence of an effect is further illustrated by noting that in the absence of an effect (i.e. when the p-value is 1) observed power is equal to the employed alpha threshold (e.g. 0.05). Hence, rather counterintuitively, low power may actually argue for the absence of an effect. Because power can only be calculated assuming the null-hypothesis is false, this metric cannot be used to make claims in favour of the null-hypothesis. Furthermore, as discussed in the preceding section, statistical tests cannot be used to support the strict null-hypothesis, as such this also holds for derived metrics such as p-value and power. While power remains essential when designing a future study, it should not be used to interpret results of a completed study. At this stage more relevant metrics such as confidence intervals are available which do not condition on the presence or absence of an effect, and provide information on accuracy as well as on effect magnitude. *“

**I do not understand “Finally, depending on the area of research overlooked, true positive results may be more harmful than false positive results”. Is meant that setting too stringent boundaries for significance can be harmful? Consider rephrasing to make its meaning clear.**

**Response: **The following has been added to clarify this matter further.

 Page 9

 “

*Finally, while decreasing the significance threshold (e.g. from 0.05 to 0.005) decreases the type 1 error rate this decreases power as well, and hence may decrease the number of true associations discovered. Depending on the area of research overlooked, true positive results may be more harmful than false positive results. For example, protein drug targets identified in early drug development are often subjected to a substantial number of follow-up analyses, which filter out false positive results. Such follow-up studies, however, rarely expand the number of candidates, hence suggesting a more inclusive approach might be more considerate. In settings more proximal to clinical implementation and less discovery oriented, such as phase 3 clinical trials, stringent multiple testing correction is clearly called for.*

 “

# Reviewer report: Reviewer response for version 1

**DOI:** 10.5256/f1000research.174018.r350489

**Published:** 2025-01-02

**Authors:** Ying Cui

**Affiliations:** 1 Stanford University, Stanford,, California, USA

The author have made very interesting points on common mistakes when interpreting results from statistical tests that fail to reject the null hypothesis. There are some minor comments as listed below:

 1. In Page 5, it was mentioned that "Hence, a more relevant alternative to post-hoc power calculations is to evaluate the extent to which the CI includes clinically relevant effect estimates, which is in line with the aforementioned equivalence/non-inferiority approach.". It can be helpful if the author could provide a specific example (e.g. further discussion with the two HR estimates using equivalence/non-inferiority approach) to further illustrate this.

 2. In the last line of Page 5, "naïve" should be "naive".

 3. In the last lines of Page 7, it was recommended that "researchers should routinely apply composite null hypothesis tests evaluated against meaningful bounds of

 insignificance". It would be helpful if the author could provide more discussion with suggestions on how to identify the "bounds of insignificance".

Is the topic of the opinion article discussed accurately in the context of the current literature?

Yes

Are arguments sufficiently supported by evidence from the published literature?

Yes

Are all factual statements correct and adequately supported by citations?

Yes

Are the conclusions drawn balanced and justified on the basis of the presented arguments?

Yes

Reviewer Expertise:

Hypothesis testing, Biostatistics

I confirm that I have read this submission and believe that I have an appropriate level of expertise to confirm that it is of an acceptable scientific standard.

# Response

**Published:** 2025-02-04

**Authors:** Amand Schmidt

**Affiliations:** University College London Faculty of Population Health Sciences, London, England, UK

**Reviewer Comments:**

 The author have made very interesting points on common mistakes when interpreting results from statistical tests that fail to reject the null hypothesis. There are some minor comments as listed below:

 1. In Page 5, it was mentioned that "Hence, a more relevant alternative to post-hoc power calculations is to evaluate the extent to which the CI includes clinically relevant effect estimates, which is in line with the aforementioned equivalence/non-inferiority approach.". It can be helpful if the author could provide a specific example (e.g. further discussion with the two HR estimates using equivalence/non-inferiority approach) to further illustrate this.

**Response: **Thank you, we have included the following example, using the VOYAGER PAD trial results.

 Page 6

*“*

*For example, the VOYAGER PAD HR estimate of 0.86 (95%CI 0.40;1.87) for bleeding risk in people with endovascular PAD clearly shows that the collected data supports a wide range of effect estimates, including potentially harmful associations. However, because the confidence interval only partially overlap with the proposed (hypothetical) upper bounds of acceptable harm of 1.25, testing against this bound results a p-value of 0.17 which is considerably smaller than testing against the complete absence of an effect: p-value 0.70. By comparison, the observed power estimate for these results is 7%, which implies that if the true HR was 0.86 one would have rejected the strict null-hypothesis in 7 out of 100 repeated experiment. As such observed power provide limit information relative to the presented alternative approaches, particularly the confidence interval based approach which allows for an informative discussion of benefits and harms in terms of effect magnitude(s).*

*“*

**Reviewer Comments:**

 2. In the last line of Page 5, "naïve" should be "naive".

**Response:** Thank you.

**Reviewer Comments:**

 3. In the last lines of Page 7, it was recommended that "researchers should routinely apply composite null hypothesis tests evaluated against meaningful bounds of

 insignificance". It would be helpful if the author could provide more discussion with suggestions on how to identify the "bounds of insignificance".

**Response: **The following has been included on page 4

 “

*Defining bounds of equivalence or non-inferiority is challenging and a possible source of contention. Typically, such bounds are defined by combining statistical and clinical considerations. For example, evidence from previous studies can be meta-analysed to obtain a pooled effect estimate and confidence interval, where the confidence interval limits can be multiplied by a constants representing the amount of effect that one would like to preserve or rule out (for safety). ^7^*

 “
